# Stricture Ilei, Laparo-enterotomy*Read before the Buffalo Medical Association.

**Published:** 1880-03

**Authors:** Herman Mynter


### STRICTURA ILEI, LAPARO-ENTEROTOMY.*

REPORTED BY HERMAN MYNTER, M. D.

Mr. M., 34 years of age; father is living; mother died in his childhood; has had healthy children; has not had venereal disease; always enjoyed good health; never suffered from any trouble of the bowels. His disease commenced about ten months ago, after having been exposed to inclement weather on a country-road; he was thoroughly chilled, which was followed by a severe pain in the abdomen, to the left of the naval. By the use of hot fomentations, rest and appropriate medication, the pain disappeared. Three weeks later the pain returned with hiccough and eructations. For several months the attacks returned at intervals of fourteen days, and were accompanied with meteorrhismus, borborygm and profuse vomiting, sometimes several quarts being vomited up. The borborygm increased in course of time, and at last became continuous. During his whole disease he suffered from constipation; cathartics generally increased the pain and vomiting; he diminished in weight about thirty pounds in half a year.

He consulted me the first time in October, 1879; he stated that the attacks now came on about every ten days, and were always accompanied with profuse vomiting, which gave great relief. The substance vomited was a sour fluid, followed by a yellow, extremely bitter fluid, and at last feculent matter. The

*Read before the Buffalo Medical Association.

borborygm was now always present, occurring every few minutes, and could be heard many feet away.

The abdomen was found slightly enlarged and tympanitic; enormous peristaltic movements were apparent through the abdominal wall; every few minutes a part of the bowels would become distended, and protrude as a tumor as large as a fist, and then slowly disappear with a loud gurgling sound. Most frequently it took place in the left iliac region. No tumor could be felt, and there was no pain on pressure. The examination of the rectum did not reveal anything abnormal; all other organs were healthy. My diagnosis was an internal constriction of unknown character. I advised discontinuance of all cathartics, absolute rest during the attacks, together with the administration of opium, appropriate diet and tincture of belladonna, (five drops four times a day). For two weeks he felt better under this treatment. I lost track of him until January 19th, 1880. He stated that during November and December the attacks increased in frequency; the vomiting occurred after almost every meal, and sometimes in enormous quantities, and of feculent character; he had occasionally evacuation of the bowels of soft character, without blood; the borborygm was constantly present; he had kept his bed for six weeks, and was now in a state of extreme starvation, with pale, cachectic color. The abdomen was so retracted, that the corpora vertebrarum and the pulsating aorta were plainly visible. The same abnormal peristaltic movements were seen, especially in left inguinal region, where a slight prominence of doubtful character was felt. The same gurgling sound was heard every few minutes, preceded by partial distension of the bowels. The patient, his family and friends, were aware of impending death, the attending physicians having declared the case hopelessly fatal.

After a consultation with Dr. Miner, who concurred in the diagnosis of internal stricture of unknown character, and considered, under existing circumstances, operative measures justifiable, and after explaining the nature and dangers of the operation to the patient and his friends, operation was determined upon. For three days pancreatic meat-solution was injected into the rectum, five ounces of meat being used every three hours, with ten grains of pure pancreatine. The injections were all retained and the patient gained in strength; five drops tincture belladonna were given every four hours; a movement from the bowels occurred the next day, of soft but natural character. The most important question, the seat of the stricture, could not be fully determined. A large-sized rectal bougie could be introduced its full length in the rectum, and felt in the left iliac region. The entire descending colon could be filled with water by injection, showing that the stricture was not near the rectum. Taking into consideration, that an almost natural movement of the bowels occasionally occurred, while the gurgling sound through the stricture indicated that only very thin fluids could pass the obstriction, I concluded that the stricture was far up, possibly near the cœcum, or in the ileum. If near the rectum, the patient in all probability would have had continuous diarrhœa, as the thin fluids would not have been absorbed before reaching the rectum.

January 23d—Operation under chloroform, Drs. Miner, Burwell, Tobie, Hauenstein, Lothrop and McNiel being present. The incision was made in the linea alba under antiseptic precautions, from the navel down towards the symphysis, and afterwards enlarged one inch upwards to the left of the navel. In the pelvic cavity, on the right side, a hard, solid stricture was found and brought out through the wound. It was about one inch in length, ring-formed, contracted, hard as cartilage; above the stricture the bowels were very much dilated, and the walls hyperthrophied; below the stricture the bowels were collapsed and the walls thin and normal. The stricture had its place in the jejunum or ileum, where could not be ascertained. As the establishment of an artificial anus would be scarcely less dangerous, and in this case, if it should terminate in recovery, which was in the range of possibilities, would always be permanent, and as the resection of the constricted part would be a very difficult proceeding, as one end was enormously dilated, the other very contracted, we resolved to establish a fistula between the bowels above and below the stricture. This was done about two inches from the stricture, the bowels being brought in parallel position, and first sewed together with six carbolized silk sutures for about one inch. On each side of and near to these sutures an incision was made in the bowels about three-fourths of an inch long, and the entire edges of the wounds were sewed together in a similar manner. The knots of the first sutures were in that way inside the bowels, the rest outside. In this manner the peritoneum was at every spot united with peritoneum.

The peritoneal cavity was well cleaned, the bowels reposited, and the wound united with six deep and several superficial carbolized silk sutures, and antiseptic bandage applied. The patient was very low after the operation, but towards evening had normal temperature and a rather weak pulse of 96. Opium in 20 drops doses was given every two hours; the following morning he felt comfortable, had no pain or tenderness in the abdomen; no meteorrhismus or vomiting; the borborygm had perfectly disappeared; the bowels moved; urine was freely evacuated. Pancreatic meat solution with whisky were given per rectum. He felt well till towards evening, when the pulse grew weaker and weaker, and he died about thirty hours after the operation from exhaustion.

At the post mortem examination, twenty-four hours after death, the wound in the abdominal wall was found partially agglutinated.

No symptoms of peritonitis present; the stricture was found about eight to ten feet from the pylorus, twelve to fourteen feet from the caecum. The stricture was formed by a circular ulceration, and did not admit the tip of the finger; no swelling of glands in the mesentery. The fistula was in such exact opposition, that when water, with a powerful syringe, was thrown into the bowels above the stricture, it passed the fistula without a drop coming out between the sutures.

